# Identification and Validation of Reference Genes for Seashore Paspalum Response to Abiotic Stresses

**DOI:** 10.3390/ijms18061322

**Published:** 2017-06-21

**Authors:** Yu Liu, Jun Liu, Lei Xu, Hui Lai, Yu Chen, Zhimin Yang, Bingru Huang

**Affiliations:** 1 College of Agro-grassland Science, Nanjing Agricultural University, Nanjing 210095, China; 2016220004@njau.edu.cn (Y.L.); liujun825@njau.edu.cn (J.L.); 2015220002@njau.edu.cn (L.X.); 2016120003@njau.edu.cn (H.L.); nauyzm@njau.edu.cn (Z.Y.); 2 Department of Plant Biology and Pathology, Rutgers University, New Brunswick, NJ 08901, USA

**Keywords:** seashore paspalum, quantitative real-time polymerase chain reaction (qRT-PCR), reference gene, abiotic stress

## Abstract

Seashore paspalum (*Paspalum vaginatum*) is among the most salt- and cadmium-tolerant warm-season perennial grass species widely used as turf or forage. The objective of this study was to select stable reference genes for quantitative real-time polymerase chain reaction (qRT-PCR) analysis of seashore paspalum in response to four abiotic stresses. The stability of 12 potential reference genes was evaluated by four programs (geNorm, NormFinder, BestKeeper, and RefFinder). *U2AF* combined with glyceraldehyde-3-phosphate dehydrogenase (GAPDH) showed stable expression in Cd-treated leaves and cold-treated roots. *U2AF* and *FBOX* were the most stable reference genes in Cd-treated roots and cold-treated leaves. In Polyethylene Glycol (PEG)- or salt-treated roots, the reference gene *U2AF* paired with either *ACT* or *CYP* were stable. *SAND* and *CACS* exhibited the most stability in salt-treated leaves, and combining *UPL*, *PP2A*, and *EF1a* was most suitable for PEG-treated leaves. The stability of *U2AF* and instability of *UPL* and *TUB* was validated by analyzing the expression levels of four target genes (*MT2a*, *VP1*, *PIP1*, and *Cor413*), and were shown to be capable of detecting subtle changes in expression levels of the target genes in seashore paspalum. This study demonstrated that *FBOX*, *U2AF*, and *PP2A* could be used in future molecular studies that aim to understand the mechanisms of abiotic stress tolerance in seashore paspalum.

## 1. Introduction

Quantifying the level of gene expression is a critical step for gene discovery and molecular analysis [1]. Quantitative real-time polymerase chain reaction (qRT-PCR) is commonly used and regarded as a highly effective method for measuring gene expression levels across different tissues, developmental periods, and biotic or abiotic stress conditions [2]. However, the accuracy of qRT-PCR analysis is dependent on the stability of reference genes, the quantity and purity of mRNA templates, the enzymatic efficiency in cDNA synthesis, and the efficacy of PCR amplification [3]. Among those factors, the stability of reference genes is likely a key factor in controlling the precision of qRT-PCR results.

A number of reference genes have been screened from many plant species, and the specific reference genes suitable for gene expression quantification vary with plant species [4,5], as a result of their having different response mechanisms. These traditional reference genes include those associated with primary metabolism or other cellular processes, such as actin (ACT), tubulin (TUB), elongation factor 1a (EF1a), glyceraldehyde-3-phosphate dehydrogenase (GAPDH), and 18s ribosomal RNA (18S rRNA) [6,7]. However, recent studies have found that some of these commonly used reference genes present unstable expression in different plant species, tissues, or environmental conditions. For example, ACT was unstable under salinity, drought, cold, and heat stress in Bermuda grass (*Cynodon dactylon*) [8] but had stable expression in Kentucky bluegrass (*Poa pratensis*) [4]. The expression of GAPDH and TUB in Peking willow (*Salix matsudana*) showed large variations under drought and salt treatments [9]. Therefore, microarray and transcriptome data were used to develop new reference genes with highly stable expression levels, such as SAND family protein (SAND), F-box/kelch-repeat protein (F-box), clathrin adapter complex subunit family protein (CACS), splicing factor (U2AF), and TIP41-like family protein (TIP41), all of which were identified in *Arabidopsis thaliana* [10]. Homologous genes of the new reference genes listed above were identified in other species following Basic Local Alignment Search Tool (BLAST) alignment analyses of transcriptome and EST data. Results of other studies showed that CACS of buckwheat (*Fagopyrum esculentum*) and cork oak (*Quercus suber*) and TIP41 and SAND of *Caragana intermedia* were demonstrated to have stable expression under different experimental conditions [11,12,13]. CYP and U2AF were shown to be the most stable genes in different tissues of switchgrass (*Panicum virgatum*) under different stress conditions [14]. The previous work strongly suggests that it is important to select suitable reference genes for different organs in specific plant species under various environmental conditions in order to accurately quantify expression levels of target genes using qRT-PCR.

Seashore paspalum (*Paspalum vaginatum*) is a widely used forage and turfgrass species with a broad range of variability in stress tolerance genes, particularly those associated with salinity and cadmium tolerance [15]. Identification of stable reference genes under different environmental conditions is imperative for efficient molecular breeding and discovery of stress-related genes in seashore paspalum. The objective of this study was to identify stable reference genes for qRT-PCR analysis of target-gene expression levels in leaves and roots of seashore paspalum under salinity, drought, cold, and heat stress. The expression levels of four target genes (*MT2a*, *VP1*, *PIP1*, and *Cor413*) isolated from seashore paspalum were used to validate the effectiveness of the selected genes identified in the study as references. According to homolog comparison between seashore paspalum transcriptome data and Arabidopsis microarray data, 12 candidate reference genes, including the five traditional genes (*EF1a*, *ACT*, *GAPDH*, *TUB*, and *UPL*) and the seven new genes selected from *Arabidopsis* (*SAND*, *CACS*, *FBOX*, *PP2A*, *CYP*, *U2AF*, and *TIP41*), were examined in this study.

## 2. Results

### 2.1. Identification of PCR Amplicons, Primer Specificity, and Amplification Efficiency of qRT-PCR

**Figure 1:**
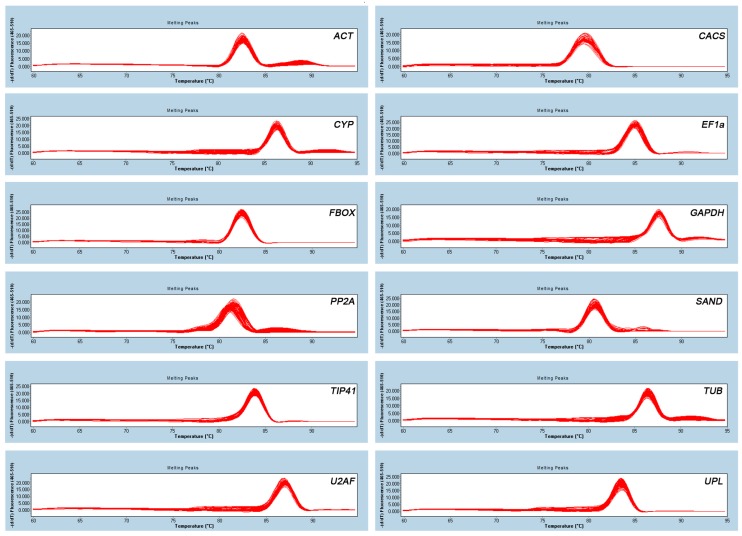
Primer specificity. Melting curves of 12 genes (*EF1*α*a*, *CACS*, *GAPDH*, *TIP41*, *SAND*, *ACT*, *TUB*, *PP2A*, *FBOX*, *UPL*, *CYP*, and *U2AF*) showing single peaks.

The names, primer sequences, and amplicon lengths of the 12 reference genes are given in Table 1. Primer specificities were confirmed by analyzing melting curve assays of qRT-PCR amplicons that produced a single peak (Figure 1). The sequences of PCR amplicons were nearly identical (97–100% in similarity) to the corresponding transcriptome data of seashore paspalum (data not shown). qRT-PCR efficiencies measured by the LinRegPCR software (Version 2012.0) for all 12 genes varied from 1.89 to 1.98 (Table 2), indicating acceptable efficiency (1.8 ≤ E ≤ 2) [16].

### 2.2. Expression Levels and Variations of Reference Genes

**Figure 2:**
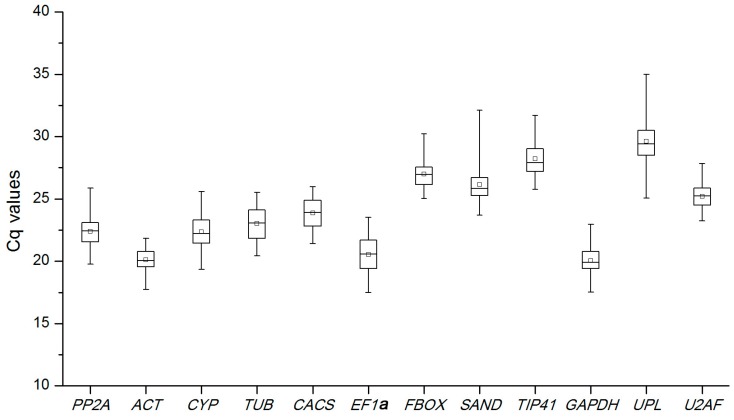
The quantification cycle (*C*q) values of the 12 candidate reference genes across all samples under four abiotic stresses. Lines across the box plot of *C*q value represent the median values. Lower and upper boxes show the 25^th^ percentile to the 75^th^ percentile. Whiskers represent the maximum and minimum values.

The quantification cycle (*C*q) values of the 12 candidate reference genes were measured by qRT-PCR analysis and ranged from 18 to 32 (Figure 2), with the lower *C*q values showing higher mRNA transcript levels. Variations in each reference gene are exhibited in the box plot (Figure 1). Among the 12 candidate reference genes, UBL had the lowest expression level with a mean *C*q of 29.6, while *GAPDH* showed the highest expression level with a mean *C*q of 20.1 (Figure 2). The coefficients of variation (lower values represent higher variability) of the 12 reference genes were 3.75% (*FBOX*), 3.96% (*U2AF*), 4.15% (*ACT*), 4.42% (*TIP41*), 5.25% (*CACS*), 5.31% (*SAND*), 5.37% (*TUB*), 5.40% (*PP2A*), 5.42% (*GAPDH*), 5.46% (*UBL*), 5.79% (*CYP*), and 6.65% (*EF1*α).

### 2.3. Stability of Candidate Reference Genes

#### 2.3.1. geNorm Analysis

**Figure 3:**
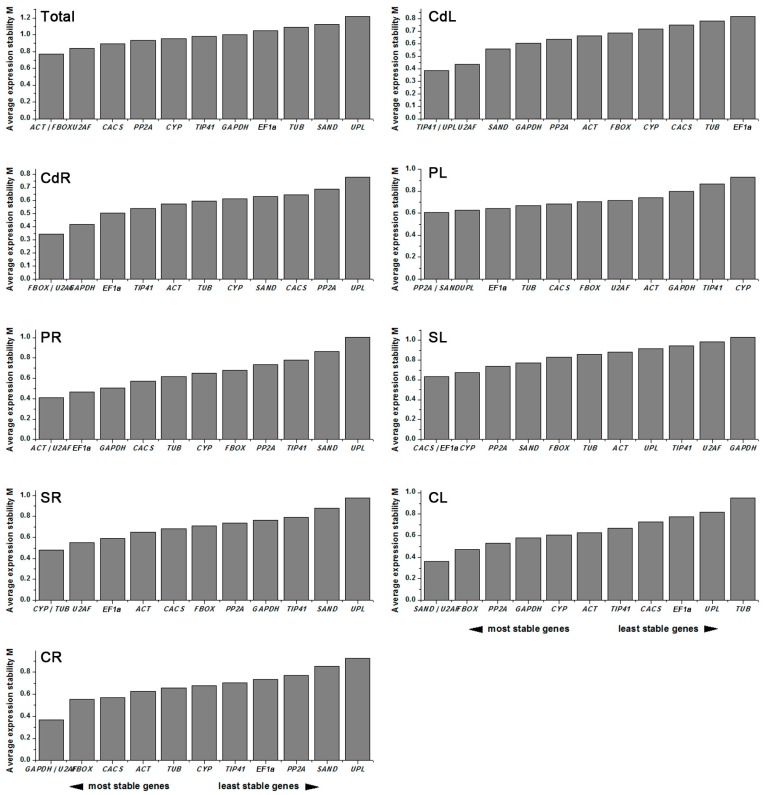
Gene expression stability values (M) and rankings of 12 reference genes as assayed by geNorm. The most stable genes are on the left and the least stable genes are on the right.

The M values calculated by geNorm software (V3.5, Ghent, Belgium), were applied to evaluate the stability of reference genes by comparing the average variation of a gene to all others. The M value of 1.5 was used as a threshold for expression stability, and M values lower than 1.5 indicated higher stability. Based on this principle, it was determined that out of all samples, *ACT* and *FBOX* from both leaf and root tissues had the same M values and were the two most stable reference genes for all stress treatments. Similarly, stabilities of reference genes were evaluated, including *TIP41*/*UPL* in cadmium-treated leaves (CdL), *FBOX*/*U2AF* in cadmium-treated roots (CdR), *PP2A*/*SAND* in PEG-treated leaves (PL), *ACT*/*U2AF* in PEG-treated roots (PR), CACS/EF1a in salt-treated leaves (SL), *CYP*/*TUB* in salt-treated roots (SR), *SAND*/*U2AF* in cold-treated leaves (CL), and *GAPDH*/*U2AF* in cold-treated roots (CR) (Figure 3). 

**Figure 4:**
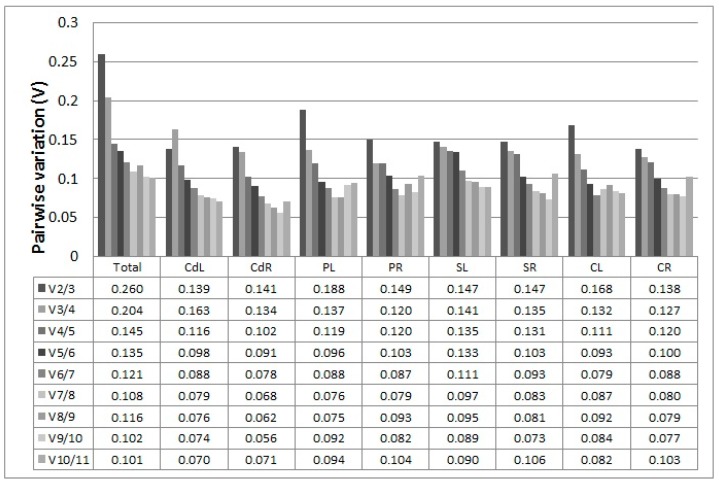
Pairwise variation (V) of the candidate reference genes calculated by geNorm. V_n_/V_n+1_ values were used to determine the optimal number of reference genes.

The geNorm program is typically used for determining the optimal number of reference genes required for accurate normalization. When a small variation appears between *V*_n/n+1_ and V_n+1_/V_n+2_ or the *V*_n/n+1_ value is lower than the threshold of 0.15, the value (*n*) can be considered as the optimal number of reference genes. The V_2/3_ values for the *CdL*, *CdR*, *PR*, *SL*, *SR*, and *CR* samples were lower than 0.15 (Figure 4), indicating that two reference genes were suitable for normalization. Three reference genes were selected after the V_3/4_ values of PL and CL samples were indicated to be below 0.15. The V_4/5_ value (0.145) of all samples showed that four genes could be useful for normalization of all the samples.

#### 2.3.2. NormFinder Analysis

Evaluation values detected by NormFinder (V0953, Aarhus, Denmark) are shown in Table 3, with lower values indicating higher stability. Among all samples, the four reference genes with the highest stability overall were *FBOX* (0.519), *ACT* (0.604), *U2AF* (0.67) and *PP2A* (0.673) (Table 3). *U2AF* and *GAPDH* were identified as the two most stable genes in *CdL* and *PR* samples, while *U2AF* and *FBOX* were ranked as having the highest stability in *CdR* and SR samples. *UPL* or *SAND* was the most stable gene in PL and SL samples. *U2AF* had the highest stability in CL and CR samples. *TUB* and *UPL* had the lowest stability rankings out of all samples except for PL.

#### 2.3.3. BestKeeper Analysis

The rankings of reference genes based on CV and SD values by BestKeeper (Version1.0, Munich, Germany) analysis are shown in Table 4, with lower SD and CV representing higher stability. *FBOX* and *U2AF* exhibited the highest stability of *CdL*, *CdR*, and PR samples. The most stable genes were *FBOX* and *CACS* for CL and CR samples, *U2AF* and *ACT* for SR samples, *PP2A* and *ACT* for SL samples, and *UPL* and *TUB* for *PL* samples.

#### 2.3.4. RefFinder Analysis

RefFinder (Version 1.0), available online: http://www.leonxie.com/referencegene.php) analysis was performed to obtain a comprehensive evaluation of candidate reference genes by integrating three common analysis programs (geNorm, Normfinder, and BestKeeper) and the ΔCq method. According to the analysis of RefFinder and geNorm (Table 5), the four most stable genes for all samples were *FBOX*, *ACT*, *U2AF* and *PP2A*, while *UPL* was the least stable reference gene. U2AF combined with different genes could be used as reference genes for *CdL*, *CdR*, *PR*, *SR*, *CL*, and *CR* samples. *UPL*, *PP2A*, and EF1a from PL samples and *SAND* and *CACS* from SL samples were suitable reference genes, while UPL presented unstable expression in total samples that included *CdR*, *PR*, *SR*, and *CR*. 

### 2.4. Detection of Four Target Gene Expression Levels Normalized by Screened Reference Genes

**Figure 5:**
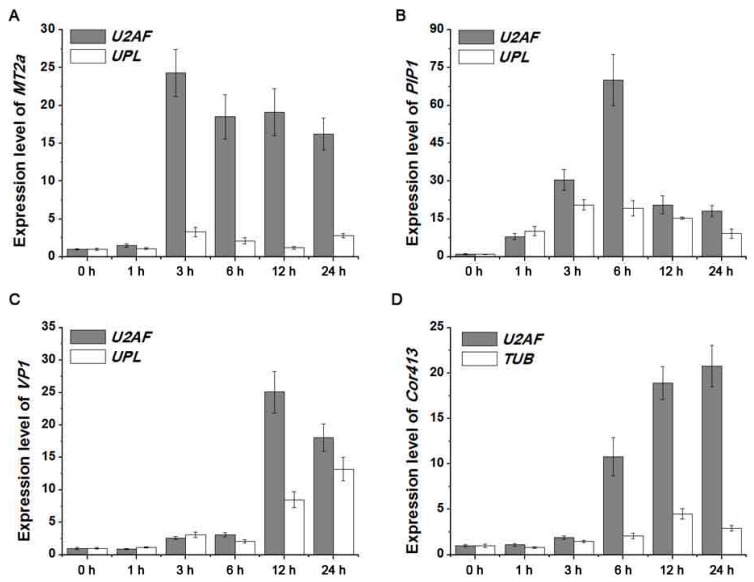
Relative expression of four target genes. (**A**) *MT2a* expression detection normalized by reference genes *U2AF* and *UPL*; (**B**) *PIP1* expression detection normalized by reference genes *U2AF* and *UPL*; (**C**) *VP1* expression detection normalized by reference genes *U2AF* and *UPL*; (**D**) *Cor413* expression detection normalized by reference genes *U2AF* and *TUB*.

To confirm the utility of the reference genes, the expression patterns of four target genes were detected (Figure 5), including *MT2a* in cadmium-treated roots (CdR) samples, *PIP1* in PEG-treated roots (PR) samples, *VP1* in salt-treated roots (SR) samples, and *Cor413* in cold-treated leaves (CL) samples. The most stable reference gene, *U2AF*, and two unstable genes, *UPL* and *TUB*, were selected for qRT-PCR analysis out of the four samples, and the results exhibited significant differences in fold changes and response timing (Figure 5). Under cadmium treatment, *MT2a* expression normalized by *U2AF* exhibited a 20-fold increase after 3 h, but showed a three-fold increase when normalized by *UPL*. The expression of *PIP1* was highest at 6 h when controlled by reference gene *U2AF* but reached the same level of expression at 3 h when normalized by *UPL*. Similar differences were also found in SR and CL samples. These results demonstrate that the accuracy of qRT-PCR analysis could be altered by the use of different reference genes.

## 3. Discussion

Previous studies have shown that there is no single reference gene that can be used for the quantification of target gene expression levels for all experimental conditions or plant species. Reference genes have been identified for several perennial grass species, including *Poa pratensis* [4], *Cynodon dactylon* [8], *Lolium perenne* [17], *Panicum virgatum* [14], *Agrostis stolonifera* [18], and *Festuca arundinacea* [19]. This study is the first to identify several reference genes suitable for qRT-PCR normalization in both leaves and roots of seashore paspalum exposed to four abiotic stresses (salinity, heavy metal cadmium, drought, and cold).

In previous studies, geNorm, NormFinder, and BestKeeper, produced different results, because each of the three software have different calculation methods [8,18]. RefFinder, a comprehensive program that integrates data from geNorm, Normfinder, BestKeeper and the Δ*C*q method, is used to screen reference genes and obtain an accurate evaluation [8,13,18]. By interpreting results from four commonly used methods (GeNorm, NormFinder, BestKeeper, and RefFinder), several stable reference genes under different conditions in seashore paspalum were identified in this study. *U2AF* was reported to serve as a stable reference gene associated with nematode inoculation in *Pinus massoniana* [20]; however, its use as a stable reference gene for abiotic stresses has not yet been reported. The current study found that *U2AF* showed stable expression in most samples with the exception of leaves under salinity and drought stress in seashore paspalum. *FBOX* was used as a stable reference gene for the normalization of cold-stressed or salicylic acid-treated rapeseed (*Brassica napus*) and for different tissues, organs, and developmental stages of aromatic litsea (*Litsea cubeba*) [21,22], as well as for cadmium-stressed soybean (*Glycine max*) samples [23]. By contrast, *FBOX* exhibited unstable expression in large leaf gentian (*Gentiana macrophylla*) leaves and roots in response to silver and copper stress [24]. *FBOX* was the most stable reference gene for total samples, cadmium-treated roots, and cold-treated leaves in this study. The expression levels of *PP2A* and *CACS* in Bermuda grass were stable in roots and leaves under salt stress, in leaves under drought stress, and in roots exposed to cold and heat stress [8]. In previous studies on PEG-treated roots of buckwheat and *C. intermedia*, *SAND* was identified as having the most stable expression following abiotic stress [11,13]. The current study indicated that *SAND* and *CACS* in salt-treated leaves and *PP2A* in PEG- and cold-treated leaves exhibited stable expression. 

It is particularly interesting to note that several reference genes exhibited differential expression patterns in seashore paspalum with respect to other grass species under same types of abiotic stresses. It was previously reported that *UPL* was the most stable reference gene in salt-treated roots and cold-treated roots of Bermuda grass and creeping bentgrass [8,18]; however, *UPL* exhibited the most unstable expression in salt-treated roots and cold-treated roots of seashore paspalum in this study. Several other widely used reference genes, including *TUB*, *GAPDH*, and *EF1α*, have been utilized for gene expression normalization in different plant species, but expression patterns in different species under different environmental conditions are variable [12,13,23]. EF1α and TUB showed the most stable expression under salinity and drought stress in soybean and black gram (*Vigna mungo*) [25,26] and under cold treatment in desert poplar (*Populus euphratica*) [27]. In this study, however, stabilities of *EF1α* and *TUB* were lower than those of several other reference genes under the four abiotic stresses. *GAPDH* was a more stable reference gene for PEG-treated leaves of buffalo grass (*Buchloe dactyloides*) [28], but it exhibited unstable expression in rice (*Oryza sativa*) under various environmental conditions [29]. In the current study, *GAPDH* showed stable expression in cadmium-treated leaves but unstable expression in salt-treated leaves. The results from this study, when compared to those produced previously by others, suggest that unique reference genes should be used for the accurate quantification of gene expression in seashore paspalum. 

Expression levels of target genes were found to vary significantly when normalized using stable and unstable reference genes, which led to misinterpretation of experimental results. In this study, stabilities of reference genes were further validated by examining the expression patterns of four target genes. The results showed that expression patterns of the target genes in response to cadmium, salt, drought, and cold stress were variable due to the use of different references genes, indicating the importance of internal control genes for qRT-PCR analysis. The stable reference genes identified and validated in this study have provided accurate qRT-PCR results that may be used for target gene expression of seashore paspalum under different abiotic stresses in the future. Findings from this study will help facilitate the identification of stress-responsive genes and molecular mechanisms conferring stress tolerance to seashore paspalum in future work. The current results furthermore provide suitable resources for qRT-PCR analysis in other species closely related to seashore paspalum.

## 4. Materials and Methods

### 4.1. Plant Materials and Stress Treatments

Seashore paspalum (cv. ‘SeaIsle 2000’) was collected from field plots located at the Grass Research Centre of Nanjing Agricultural University in Nanjing, China. Stolons measuring 4–5 cm in length and having two nodes were hydroponically cultivated for 10 days in half strength Hoagland’s nutrient solution in a controlled-climate growth chamber (MT8070iE, shoreline Technology, Xubang, Jinan, China) with 12 h photoperiod (850 µmol photons m^−2^·s^−1^), 28/25 °C (day/night) and 60% relative humidity. Seedlings were transferred to nutrient solution containing 250 mM NaCl for salinity treatment, 1 mM cadmium for heavy metal treatment, or 20% PEG6000 for drought treatment. Cold stress was imposed at 3 °C in an incubator (Haier, Qingdao, China). Each treatment was performed using three biological replicates having three plants in each replicate. Leaves and roots were separately collected at 0, 1, 3, 6, 12, and 24 h of each treatment, and the tissue was immediately frozen in liquid nitrogen and stored at −80 °C for further analysis.

### 4.2. Total RNA Isolation and cDNA Synthesis

Total RNA was isolated according to the RNAiso kit (TaKaRa, Dalian, China) and was then treated with RNase-free DNaseI (TaKaRa). RNA concentration was detected spectrophotometrically (NanoDrop 2000, Thermo, Waltham, MA, USA) at wavelengths of 230, 260, and 280 nm, and the 260/280 nm ratio within the range of 1.80–2.20 and 260/230 nm ratio at approximately 2.00 were obtained. First-strand cDNA was synthesized based on 0.5 μg total RNA using the M-MLV reverse transcription system (TaKaRa), according to the manufacturer’s instructions. The cDNAs were diluted in a 1:20 ratio of *CDNA* to nuclease-free water prior to the qRT-PCR analyses.

### 4.3. Selection of Reference Genes and Primer Design

Arabidopsis nucleotide sequences from the potential reference genes served as query sequences for a TBLASTX search of the seashore paspalum transcriptome database (unpublished). Twelve candidate reference genes (*EF1a*, *ACT*, *GAPDH*, *TUB*, *UPL*, *SAND*, *CACS*, *F-box*, *PP2A*, *CYP*, *U2AF*, and *TIP41*) were identified and corresponding NCBI accession numbers and gene ontologies are given in Table 1. Specific primers for qRT-PCR were designed using Primer Premier 5.0 software to have melting temperatures between 55–65 °C, primer lengths between 19–24 bp, and amplicon lengths between 100–300 bp (Table 1).

### 4.4. qRT-PCR Analysis

The qRT-PCR procedure was performed using the LightCycler 480 SYBR Green I Master reaction system with a LightCycler 480 II instrument (Roche, Basel, Switzerland). Each 15-μL reaction mixture consisted of 7.5 μL of 2× concentrated SYBR Green I Master Mix, 5 μL of diluted cDNA, 0.4 μL of each primer (10 µM total), and 1.7 µL double-distilled water. The reaction conditions included an initial denaturation step at 95 °C for 10 min followed by 45 cycles of 95 °C for 15 s, 58 °C for 15 s, and 72 °C for 30 s, after which a melt curve was produced at 60–95 °C. Each qRT-PCR analysis was performed in triplicate.

### 4.5. Stability Analysis

Amplification efficiencies of each qRT-PCR were calculated by the slope of the line (E = 10^slope^), with the software LinRegPCR, based on Log (fluorescence) per cycle number data as an assumption-free method to calculate starting concentrations of mRNAs, which is available on request [30]. The stability of reference genes was determined with four programs, including GeNorm [1], NormFinder [31], BestKeeper [32] and RefFinder (available online: http://www.leonxie.com/referencegene.php). For GeNorm and NormFinder analysis, quantification cycle (*C*q) values were converted into relative quantities using the formula 2^−Δ*C*q^, in which Δ*C*q = each corresponding *C*q value-minimum *C*q value. The expression stability measurement (M) was determined by the GeNorm program based on the average variations of a particular gene against all the other control genes in their expression levels. Through the NormFinder program, the stability value represented inter- and intra-group variation and lowest stability was ranked highest. The BestKeeper program was applied to measure the comparisons of the coefficient of variance (CV) and the standard deviation (SD), and the lowest SD and CV were used as detection indexes for the most stable reference genes. RefFinder was used to make a comprehensive analysis based on the data from GeNorm (M values), NormFinder (Stability values), BestKeeper (CV and SD), and Δ*C*q values.

### 4.6. Validation of Reference Genes by Expression Analysis of Four Stress-Related Genes under Abiotic Stresses

Previous reports showed that MT2a, VP1, PIP1, and Cor413 were responsive to various abiotic stresses [33,34,35,36]. The four homologs MT2a, VP1, PIP1, and Cor413 (Genbank accession numbers shown in Table 1) from seashore paspalum were obtained from the transcriptome data (unpublished). For the validation of selected reference genes from qRT-PCR data, the expression levels of these four genes were analyzed using the most stable and highly varying reference genes under different treatments, calculated using the 2^−ΔΔ*C*q^ method. Three technical replicates were performed for each biological sample.

### 4.7. Statistical Analysis

A one-way analysis of variance (ANOVA) was performed to calculate whether treatment means were statistically different from one another (*p* = 0.05) using the SPSS v13.0 software (IBM, Chicago, IL, USA). 

## 5. Conclusions

This study provides the first systematic study for screening stable reference genes for use as the internal control in qRT-PCR analysis in leaves and roots of seashore paspalum under four different abiotic stresses. *FBOX*, *U2AF*, and *PP2A* could be applied as stable reference genes in future molecular studies that aim to understand the mechanisms of abiotic stress tolerance in seashore paspalum.

## Figures and Tables

**Table 1:** Reference genes and primer sequences.

Gene Symbol	Gene Name	GenBank Accession	Arabidopsis Homolog Locus	5′-Primer Sequences (Forward/Reverse)-3′	Amplicon Length (bp)
*EF1a*	*Elongation factor 1a*	KU049721	AT5G60390	GCGGACTGTGCTGTGCTTATC/AGTGGTGGCATCCATCTTGTT	153
*CACS*	*Clathrin adaptor complex subunit*	KX268090	AT5G46630	CACTGTCGAGTGGGTTCGCTAC/GCCGATGAATTTTACTTGTTGC	109
*GAPDH*	*Glyceraldehyde 3-phosphate dehydrogenase*	KX268091	AT1G13440	GTCGCATGGTACGACAACGAGT/ACGGAAAACAAAAGGCAACTCA	221
*TIP41*	*TIP41-like family protein*	KX268092	AT4G34270	TGATGAGATTGAGGGATACTCG/TACAGACGGTGGTCACCTTTGG	244
*SAND*	*SAND family protein*	KX268093	AT2G28390	CGGGGATTATGTTCTATTTTGC/TTATGGTACTGCCTGTGTCGGT	266
*ACT*	*Actin 7*	KX268094	AT5G09810	CTTCTCTCAGCACTTTCCAACA/AAACATAACCTGCAATCTCTCC	162
*TUB*	*Alpha Tubulin*	KX268095	AT5G19780	GTCGGTGAGGGTATGGAGGAAG/ATGGAAACACACAGCAGCAGTT	237
*PP2A*	*Protein phosphatase 2A*	KX268096	AT1G13320	TAAGGTACTACGCAAACCAAGC/CAACACAATACATACACAGCACACA	289
*FBOX*	*F-box/kelch-repeat protein*	KX268097	AT5G15710	GTGCTAGCCAGCTCTGCAATAG/ACACATCCGACATCAACGATTC	184
*UPL*	*E3 ubiquitin protein ligase*	KX268098	AT3G53090	TACTTGGATTCAAATACCTACAGCC/TTAGAACCCCAGAAACACCGCT	250
*CYP*	*Cyclophilin*	KX268099	AT2G29960	CTGGAAGAGATACAAACGGATC/GCCACTAATGACAGTTATAGAACG	275
*U2AF*	*Splicing factor U2af*	KX268100	AT5G42820	AGGAGCCCAGTCAGGGAAA/CACGCAGAATAGCAACTCAAAT	190
*MT2a*	*Metallothionein2a*	KX268101	AT3G09390	CAGACTCTCGTCATGGGCGT/TCTCATCGGATCAGGTAGCA	247
*VP1*	*vacuolar H^+^-pyrophosphatase 1*	KX268102	AT1G15690	GTCCCTCAACATCCTCATCAAG/TAAGTCTAAGGTAACGCCTCCA	281
*PIP1*	*plasma membrane intrinsic protein 1*	KX268103	AT4G00430	AGGGCCATCCCGTTCAAGAG/ATAACAGCGGCGGCATATTA	239
*Cor413*	*cold-regulated 413 plasma membrane protein*	KX268104	AT3G50830	TCAGGAACGCCTTCAGGAAG/GGATGGCAGAGGAGCACACT	134

**Table 2:** Amplification efficiency of qRT-PCR for 12 reference genes.

Gene	CdL	CdR	PL	PR	SL	SR	CL	CR
*ACT*	1.93 ± 0.01	1.96 ± 0.03	1.95 ± 0.02	1.93 ± 0.02	1.97 ± 0.02	1.92 ± 0.02	1.95 ± 0.03	1.94 ± 0.01
*CACS*	1.94 ± 0.02	1.97 ± 0.02	1.93 ± 0.02	1.92 ± 0.02	1.96 ± 0.02	1.96 ± 0.01	1.92 ± 0.02	1.97 ± 0.02
*EF1α*	1.98 ± 0.01	1.93 ± 0.01	1.96 ± 0.03	1.93 ± 0.01	1.94 ± 0.04	1.94 ± 0.02	1.96 ± 0.02	1.95 ± 0.03
*FBOX*	1.93 ± 0.02	1.94 ± 0.03	1.97 ± 0.03	1.96 ± 0.02	1.95 ± 0.02	1.93 ± 0.02	1.94 ± 0.02	1.93 ± 0.02
*GADPH*	1.91 ± 0.02	1.95 ± 0.02	1.93 ± 0.02	1.96 ± 0.02	1.92 ± 0.02	1.94 ± 0.03	1.91 ± 0.02	1.97 ± 0.02
*UPL*	1.93 ± 0.02	1.95 ± 0.02	1.95 ± 0.03	1.97 ± 0.02	1.95 ± 0.03	1.92 ± 0.02	1.94 ± 0.03	1.93 ± 0.03
*SAND*	1.94 ± 0.02	1.95 ± 0.02	1.94 ± 0.01	1.96 ± 0.03	1.93 ± 0.01	1.95 ± 0.02	1.94 ± 0.02	1.96 ± 0.01
*TUB*	1.96 ± 0.01	1.95 ± 0.01	1.94 ± 0.02	1.97 ± 0.02	1.94 ± 0.03	1.96 ± 0.03	1.95 ± 0.03	1.97 ± 0.02
*TIP41*	1.96 ± 0.02	1.91 ± 0.02	1.90 ± 0.02	1.91 ± 0.02	1.95 ± 0.02	1.90 ± 0.03	1.93 ± 0.03	1.91 ± 0.02
*CYP*	1.93 ± 0.02	1.94 ± 0.01	1.94 ± 0.01	1.96 ± 0.03	1.93 ± 0.01	1.95 ± 0.02	1.94 ± 0.02	1.96 ± 0.01
*PP2A*	1.95 ± 0.02	1.96 ± 0.02	1.95 ± 0.02	1.96 ± 0.02	1.93 ± 0.03	1.97 ± 0.03	1.89 ± 0.03	1.96 ± 0.02
*U2AF*	1.96 ± 0.01	1.93 ± 0.01	1.89 ± 0.02	1.92 ± 0.02	1.94 ± 0.02	1.91 ± 0.03	1.91 ± 0.02	1.92 ± 0.01

CdL and CdR: cadmium-treated leaves and roots, respectively; PL and PR: PEG-treated leaves and roots, respectively; SL and SR: salt-treated leaves and roots, respectively; CL and CR: cold-treated leaves and roots, respectively.

**Table 3:** Stability analysis of reference genes assayed by NormFinder software.

Total	Stability	CdL	Stability	CdR	Stability	PL	Stability	PR	Stability	SL	Stability	SR	Stability	CL	Stability	CR	Stability
*FBOX*	0.519	U2AF	0.374	U2AF	0.223	UPL	0.391	U2AF	0.392	SAND	0.461	U2AF	0.383	U2AF	0.326	U2AF	0.294
*ACT*	0.604	GAPDH	0.444	FBOX	0.37	PP2A	0.402	GAPDH	0.397	CACS	0.54	FBOX	0.455	PP2A	0.392	TIP41	0.458
*U2AF*	0.67	ACT	0.471	TIP41	0.376	EF1α	0.466	EF1α	0.522	TUB	0.594	CYP	0.512	FBOX	0.443	GAPDH	0.465
*PP2A*	0.673	UPL	0.506	EF1α	0.441	SAND	0.474	FBOX	0.535	PP2A	0.618	TUB	0.519	GAPDH	0.48	CYP	0.525
*TIP41*	0.703	PP2A	0.529	GAPDH	0.474	TUB	0.563	TIP41	0.55	EF1α	0.669	TIP41	0.553	CYP	0.529	TUB	0.528
*CYP*	0.773	SAND	0.53	CYP	0.478	FBOX	0.583	ACT	0.592	FBOX	0.674	CACS	0.562	SAND	0.535	FBOX	0.555
*GAPDH*	0.778	TIP41	0.594	TUB	0.484	CACS	0.618	CYP	0.687	CYP	0.738	GAPDH	0.656	ACT	0.577	ACT	0.555
*CACS*	0.811	CYP	0.606	ACT	0.519	ACT	0.638	CACS	0.687	ACT	0.755	EF1α	0.657	TIP41	0.651	CACS	0.589
*SAND*	0.963	FBOX	0.619	SAND	0.527	U2AF	0.724	TUB	0.689	UPL	0.775	ACT	0.681	EF1α	0.755	PP2A	0.694
*TUB*	1.073	CACS	0.689	CACS	0.553	GAPDH	0.857	PP2A	0.73	TIP41	0.9	PP2A	0.769	CACS	0.776	EF1α	0.869
*EF1α*	1.131	TUB	0.747	PP2A	0.815	TIP41	0.953	SAND	0.999	U2AF	0.948	SAND	1.059	UPL	0.886	SAND	1.036
*UPL*	1.496	EF1α	0.833	UPL	1.163	CYP	1.09	UPL	1.612	GAPDH	1.042	UPL	1.355	TUB	1.497	UPL	1.154

Total: pooled samples from all treatments; CdL and CdR: cadmium-treated leaves and roots, respectively; PL and PR: PEG-treated leaves and roots, respectively; SL and SR: salt-treated leaves and roots, respectively; CL and CR: cold-treated leaves and roots, respectively.

**Table 4:** Stability analysis of reference genes assayed by BestKeeper software.

Rank	Total	CV ± SD	CdL	CV ± SD	CdR	CV ± SD	PL	CV ± SD	PR	CV ± SD	SL	CV ± SD	SR	CV ± SD	CL	CV ± SD	CR	CV ± SD
1	*FBOX*	2.95 ± 0.80	*FBOX*	1.61 ± 0.44	*FBOX*	1.36 ± 0.35	*UPL*	1.42 ± 0.41	*U2AF*	2.62 ± 0.67	*PP2A*	2.43 ± 0.54	*U2AF*	2.28 ± 0.55	*FBOX*	1.89 ± 0.52	*FBOX*	1.09 ± 0.28
2	*U2AF*	3.15 ± 0.79	*U2AF*	1.73 ± 0.44	*U2AF*	1.58 ± 0.38	*TUB*	1.51 ± 0.36	*FBOX*	3.67 ± 1.03	*ACT*	2.62 ± 0.53	*ACT*	2.44 ± 0.47	*CACS*	1.92 ± 0.48	*CACS*	1.62 ± 0.37
3	*ACT*	3.39 ± 0.68	*UPL*	1.85 ± 0.54	*TUB*	1.96 ± 0.42	*FBOX*	1.52 ± 0.41	*CACS*	3.75 ± 0.91	*CYP*	3.63 ± 0.81	*PP2A*	2.49 ± 0.53	*U2AF*	1.95 ± 0.51	*EF1α*	1.88 ± 0.35
4	*TIP41*	3.67 ± 1.04	*CACS*	1.89 ± 0.47	*GAPDH*	2.02 ± 0.39	*EF1α*	1.86 ± 0.41	*ACT*	3.79 ± 0.79	*TUB*	2.00 ± 0.48	*FBOX*	2.54 ± 0.67	*PP2A*	2.15 ± 0.50	*TUB*	2.13 ± 0.46
5	*SAND*	3.77 ± 0.99	*SAND*	1.92 ± 0.50	*TIP41*	2.04 ± 0.56	*SAND*	1.92 ± 0.49	*TUB*	3.81 ± 0.89	*CACS*	2.84 ± 0.69	*CACS*	2.56 ± 0.58	*TIP41*	2.32 ± 0.67	*U2AF*	2.32 ± 0.58
6	*UPL*	4.10 ± 1.22	*ACT*	1.97 ± 0.40	*CACS*	2019 ± 0.49	*U2AF*	1.95 ± 0.49	*TIP41*	4.02 ± 1.17	*EF1α*	3.46 ± 0.74	*TUB*	2.75 ± 0.61	*CYP*	2.33 ± 0.55	*ACT*	2.47 ± 0.48
7	*GAPDH*	4.18 ± 0.84	*GAPDH*	2.02 ± 0.40	*ACT*	2.44 ± 0.47	*CACS*	2.04 ± 0.49	*EF1α*	4.17 ± 0.85	*FBOX*	1.55 ± 0.42	*EF1α*	2.75 ± 0.53	*EF1α*	2.47 ± 0.54	*GAPDH*	2.78 ± 0.56
8	*PP2A*	4.32 ± 0.97	*PP2A*	2.04 ± 0.46	*PP2A*	2.46 ± 0.52	*PP2A*	2.10 ± 0.47	*GAPDH*	4.20 ± 0.90	*SAND*	1.80 ± 0.47	*CYP*	2.86 ± 0.60	*ACT*	2.56 ± 0.52	*TIP41*	2.85 ± 0.79
9	*CACS*	4.50 ± 1.07	*CYP*	2.17 ± 0.49	*EF1α*	2.54 ± 0.49	*ACT*	2.36 ± 0.48	*CYP*	4.83 ± 1.12	*TIP41*	3.45 ± 0.96	*TIP41*	3.44 ± 0.95	*SAND*	2.58 ± 0.68	*CYP*	3.13 ± 0.67
10	*TUB*	4.70 ± 1.08	*TIP41*	2.54 ± 0.72	*CYP*	2.60 ± 0.54	GAPDH	3.41 ± 0.69	*PP2A*	4.89 ± 1.16	*GAPDH*	4.66 ± 0.92	*SAND*	3.83 ± 0.99	*GAPDH*	2.96 ± 0.59	*UPL*	3.35 ± 1.01
11	*CYP*	4.74 ± 1.06	*TUB*	2.7 ± 0.66	*SAND*	2.65 ± 0.67	*TIP41*	3.58 ± 1.01	*UPL*	5.33 ± 1.67	*UPL*	2.39 ± 0.68	*GAPDH*	4.03 ± 0.79	*UPL*	3.09 ± 0.90	*PP2A*	3.63 ± 0.81
12	*EF1α*	5.74 ± 1.18	*EF1α*	3.22 ± 0.69	*UPL*	3.04 ± 0.89	*CYP*	3.89 ± 0.90	*SAND*	5.42 ± 1.49	*U2AF*	2.89 ± 0.75	*UPL*	4.08 ± 1.22	*TUB*	4.21 ± 0.97	*SAND*	4.47 ± 1.17

Total: pooled samples from all treatments; CdL and CdR: cadmium-treated leaves and roots, respectively; PL and PR: PEG-treated leaves and roots, respectively; SL and SR: salt-treated leaves and roots, respectively; CL and CR: cold-treated leaves and roots, respectively.

**Table 5:** Most stable and least stable combination of reference genes based on RefFinder analysis.

Experimental Treatments																	
Total		CdL		CdR		PL		PR		SL		SR		CL		CR	
Most	Least	Most	Least	Most	Least	Most	Least	Most	Least	Most	Least	Most	Least	Most	Least	Most	Least
*FBOX*	*UPL*	*U2AF*	*EF1a*	*U2AF*	*UPL*	*UPL*	*CYP*	*U2AF*	*UPL*	*SAND*	*GAPDH*	*U2AF*	*UPL*	*U2AF*	*TUB*	*U2AF*	*UPL*
*ACT*		*GAPDH*		*FBOX*		*PP2A*		*ACT*		*CACS*		*CYP*		*PP2A*		*GAPDH*	
*U2AF*						*EF1a*								*FBOX*			
*PP2A*																	
